# The Stomatin-Like Protein SLP-1 and Cdk2 Interact with the F-Box Protein Fbw7-γ

**DOI:** 10.1371/journal.pone.0047736

**Published:** 2012-10-17

**Authors:** Wei Zhang, Elizabeth M. MacDonald, Deanna M. Koepp

**Affiliations:** Department of Genetics, Cell Biology and Development, University of Minnesota, Minneapolis, Minnesota, United States of America; Martin-Luther-University Halle, Germany

## Abstract

Control of cellular proliferation is critical to cell viability. The F-box protein Fbw7 (hAgo/hCdc4/FBXW7) functions as a specificity factor for the Skp1-Cul1-F-box protein (SCF) ubiquitin ligase complex and targets several proteins required for cellular proliferation for ubiquitin-mediated destruction. Fbw7 exists as three splice variants but the mechanistic role of each is not entirely clear. We examined the regulation of the Fbw7-γ isoform, which has been implicated in the degradation of c-Myc. We show here that Fbw7-γ is an unstable protein and that its turnover is proteasome-dependent in transformed cells. Using a two-hybrid screen, we identified a novel interaction partner, SLP-1, which binds the N-terminal domain of Fbw7-γ. Overexpression of SLP-1 inhibits the degradation of Fbw7-γ, suggesting that this interaction can happen *in vivo*. When Fbw7-γ is stabilized by overexpression of SLP-1, c-Myc protein abundance decreases, suggesting that the SCF^Fbw7-γ^ complex maintains activity. We demonstrate that Cdk2 also binds the N-terminal domain of Fbw7-γ as well as SLP-1. Interestingly, co-expression of Cdk2 and SLP-1 does not inhibit Fbw7-γ degradation, suggesting that Cdk2 and SLP-1 may have opposing functions.

## Introduction

Ubiquitin-mediated proteolysis is critical for cellular proliferation and proteins that function in this pathway often contribute to tumorigenesis. The F-box protein Fbw7 (hAgo/hCdc4/FBXW7) functions as a specificity factor for the modular Skp1-Cul1-F-box protein (SCF) ubiqutin ligase complex. Fbw7 is a tumor suppressor and the Fbw7 locus is mutated in many human cancer cell lines and primary tumors (reviewed in [1]). In mice, the FBXW7 locus is required for viability [2], [3] but Fbxw7^+/−^ heterozygotes exhibit increased incidence of tumor formation relative to wildtype animals [4].

The SCF^Fbw7^ complex targets a number of proteins required for cellular proliferation for ubiquitination and subsequent degradation by the proteasome, including cyclin E, c-Jun, c-Myc, mTOR, Notch, PGC1α, and SREBP [5]–[17]. The domain structure of Fbw7 includes the conserved F-box domain, required to bind Skp1 for SCF complex assembly, and eight WD40 repeats in the C-terminus [5]–[7]. The interaction of Fbw7 with its substrates is mediated through a phosphodegron motif first identified in cyclin E [18]. Structural studies show that conserved arginine residues in the WD40 repeat region of both Fbw7 as well as Cdc4, the yeast ortholog of Fbw7, are important for binding to the phosphodegron motif [19], [20]. The N-terminus of Fbw7 contains residues important for cellular localization [12], [21], but is not well studied.

Fbw7 is conserved from yeast to humans, but only mammals exhibit splice variants. In humans, there are three splice variants of Fbw7, α, β, and γ, which arise from the use of independent first exons [22]. Thus, each isoform has a unique N-terminus. The α isoform is widely expressed at high levels in many tissues, whereas the β and γ isoforms are expressed at high levels in brain and skeletal muscle and in low levels in many other tissues [22]. Nevertheless, the precise role and significance of each Fbw7 splice variant is not well understood. Since Fbw7-α is the most highly expressed Fbw7 variant in most tissues [22], it is widely thought that this isoform is largely responsible for the ubiquitination of most Fbw7 targets, although there is evidence indicating that Fbw7-γ may be the key isoform in specific situations. A recent study in which isoform-specific knockout cell lines were generated is consistent with the Fbw7-α is primary model [21]. By contrast, other work suggests that Fbw7-γ is specific for the ubiquitination of c-Myc [12], whereas Fbw7-α is prevented from targeting c-Myc for degradation because of the action of a de-ubiquitinating enzyme [23]. In addition, there is evidence that Fbw7-α is key to a proline isomerization step that is required for the recognition of cyclin E by Fbw7-γ. In this model, the binding of cyclin E to Fbw7-α is simply a precursor to ubiquitination via Fbw7-γ [24]. Finally, expression levels of cyclin E may also play a role in determining which Fbw7 variant is utilized [25].

To better understand the function of Fbw7-γ, we examined the regulation of this protein. We found that Fbw7-γ is an unstable protein, consistent with a recent report [21]. We show here that Fbw7-γ turnover is proteasome-dependent in transformed cells. We have identified a novel interaction partner called SLP-1 that binds the unique N-terminal domain of Fbw7-γ and inhibits its degradation when overexpressed. The abundance of c-Myc significantly decreases when SLP-1 overexpression inhibits Fbw7-γ degradation, suggesting that SLP-1 interaction with Fbw7-γ does not inhibit SCF activity. Both SLP-1 and Fbw7-γ also co-immunoprecipitate with Cdk2. Interestingly, SLP-1 overexpression cannot inhibit Fbw7-γ degradation when Cdk2 is also overexpressed, suggesting that Cdk2 and SLP-1 may have opposing regulatory roles with respect to Fbw7-γ.

## Materials and Methods

### Cell Culture and Reagents

HEK293T and Hela cells (obtained from ATCC) were maintained in Dulbecco’s Modified Eagle’s Medium (HyClone) with 10% Newborn Bovine Serum (Atlanta Biologicals), with 5% CO_2_.

### Cell Transfection and Infection

HEK293T cells were transfected with Lipofectamine2000 (Invitrogen) according to manufacturer’s instructions. 40 hours after transfection, cells were washed with PBS (137 mM NaCl, 2.7 mM KCl, 10 mM Na_2_HPO_4_, 2 mM KH_2_PO_4_) and collected.

### Generation of Expression Constructs

Fbw7 isoform constructs have been described [26]. SLP1 was cloned using EcoRI and SalI sites in the p3xFlag-CMV 7.1 vector (Sigma). Myc-tagged SLP1 and Fbw7 were cloned into pCS2+Myc vector or pcDNA3.1Myc/His vector. The deletion mutants of Fbw7-γ were cloned into p3X FLAG-CMV 7.1 expression vector (Sigma) by two-step PCR. A complete list of the constructs used in this study is shown in Table 1.

**Table 1:** Plasmids used in this study.

Name	Description	Reference
pFlag-Fbw7-β	CMV promoter, Flag-Fbw7-β	[40]
pFlag-Fbw7-γ	CMV promoter, Flag-Fbw7-γ	[40]
p3xFlag-Fbw7-γ unique	CMV promoter, Flag-Fbw7-γ residues 1–49	This study
p3xFlag-Fbw7-γ K-A	CMV promoter, Flag-Fbw7-γ K3A K6A K19A K22A K32A	This study
p3xFlag-Fbw7-γ ΔF-box	CMV promoter, Flag-Fbw7-γ Δ166–206	This study
p3xFlag-Fbw7 common	CMV promoter, Flag-Fbw7 residues 50–589	This study
pCS2+MT Fbw7-γ	CMV promoter, 6MYC-Fbw7-γ	This study
pCS2+MT Fbw7-γ Nterm	CMV promoter, 6MYC-Fbw7-γ residues 1–49	This study
p3xFlag-SLP-1	CMV promoter, Flag-SLP-1	This study
pCS2+MT-SLP-1	CMV promoter, 6MYC-SLP-1	This study
HA-Cdk2	CMV promoter, HA-Cdk2	[41]
HA-Cdk2-DN	CMV promoter, HA-Cdk2 Δ146N	[41]
pAS2-Fbw7-γ N-term	*ADH1* promoter, *GAL4*(1–147)HA-Fbw7-γ residues 1–49 *TRP1*	This study
c-Myc	CMV promoter, c-Myc	K. Conklin (U. Minnesota)

### Western Blot Analysis and Reagents

Cell lysates were prepared in NETN buffer (20 mM Tris pH 8.0, 100 mM NaCl, 1 mM EDTA, 0.5% NP-40) containing 1 mM NaF, 2.5 mM β-glycerophosphate and protease inhibitor cocktail (Roche Applied Science). Protein concentrations were determined by the Bio-Rad protein assay (Bio-Rad Laboratories, Inc). Cell lysates were resolved by SDS-PAGE and electrophoretically transferred to nitrocellulose membrane. Membranes were blocked in PBST (137 mM NaCl, 2.7 mM KCl, 10 mM Na_2_HPO_4_, 2 mM KH_2_PO_4_, 0.5% Tween-20) containing 5% milk for at least 40 minutes at room temperature. Blots were probed with primary antibodies followed by labeling with horseradish peroxidase conjugated anti-mouse or anti-rabbit secondary antibody (Jackson Immunoresearch). Following antibody incubation, blots were developed on film using an Enhanced Chemiluminescence kit (PIERCE) according to the manufacturer’s instructions. Densitometry of immunoblot bands was measured using NIH ImageJ. The primary antibodies used included: anti-Cdk2 antibody (M2, Santa Cruz), anti-Flag M2 antibody (Sigma), anti-HA (HA.11, Covance Research), anti-Myc (9E10, Covance Research), anti-GAPDH (Abcam), and anti-tubulin (a generous gift from Dr. Sean Conner).

### Two-hybrid Screen

The screen used the pretransformed HeLa cDNA library with the Matchmaker kit (Clontech) and was performed according to manufacturer’s instructions. Beta-galactosidase assays were performed as described [27].

### Co-immunoprecipitation Assays

Cells were lysed with NETN lysis buffer, and 200–500 µl of cell lysate was incubated with antibody at 4°C for 4 hours, then 20 µl of protein A/G agarose slurry (Santa Cruz Biotechnology) was added for another 2 hours or overnight. The beads were washed three times with at least 10 volumes of NETN lysis buffer before resolving by SDS-PAGE.

### Protein Stability Assays

HEK293T cells were transfected with the construct(s) of interest. 36 to 40 hours after transfection, cycloheximide (Sigma) was added to a final concentration of 20 µg/ml to stop protein synthesis. Cell extracts from each time point were analyzed by Western blotting. For proteasome inhibition, LLnL was added to the cells at 50 µg/ml (Sigma) 5 hours before the stability assay.

### Protein Fractionation Assay

2×10^6^ transfected cells were harvested and washed twice with ice-cold PBS followed by resuspension in buffer A (10 mM HEPES-K^+^ pH7.5, 10 mM KCl, 1.5 mM MgCl_2_, 0.5 mM DTT) in the presence of protease inhibitor cocktail (PIC). Cells were pelleted by spinning at 1000×rpm 5 min and lysed in ice-cold buffer A containing 0.5% NP-40 with PIC on ice for 10 min. The nuclei were pelleted at 3,000 rpm for 2 min at 4°C. The supernatant was collected as the cytoplasmic fraction. Nuclear pellets were washed with buffer A and then resuspended in buffer C (20 mM HEPES-K^+^ pH 7.9, 420 mM NaCl, 0.2 mM EDTA, 1.5 mM MgCl_2_, 0.5 mM DTT, 25% Glycerol) with PIC. Nuclei were incubated on ice for another 30 min with occasional vortex. Supernatant containing nuclear protein was collected by spinning at 14,500 rpm for 10 min at 4°C.

### Immunofluorescence Microscopy

HEK293T cells transfected with epitope-tagged expression constructs were grown on cover slides for 40 h. Cells were fixed with a 3% paraformaldehyde 2% sucrose solution for 10 min at room temperature. Cells were permeabilized in ice-cold 0.5% Triton X-100 solution (0.5% Triton X-100, 20 mM HEPES pH 7.4, 50 mM NaCl, 3 mM MgCl_2_, 300 mM sucrose) on ice for 5 min. and blocked with 1% bovine serum albumin in PBS (137 mM NaCl, 2.7 mM KCl, 10 mM Na_2_HPO_4_, 2 mM KH_2_PO_4_) at 37°C for 30 min. Cells were incubated with anti-Flag (1∶2000), anti-Myc (9E10, 1∶1000) or anti-HA (HA.11, 1∶1000) antibodies at 37°C for 30 min. followed by incubation with anti-mouse FITC-conjugated secondary antibodies (1∶5000) for 20 min at 37°C. Images were collected on a Zeiss Axioskop 2 microscope equipped with a Zeiss Axiocam R2 digital camera using Zeiss Axiovision software release 3.1 (Carl Zeiss, Thornwood, NY).

## Results

### Fbw7-γ Proteolysis is Regulated by a Unique N-terminal Domain and Cell Cycle Stage

**Figure 1:**
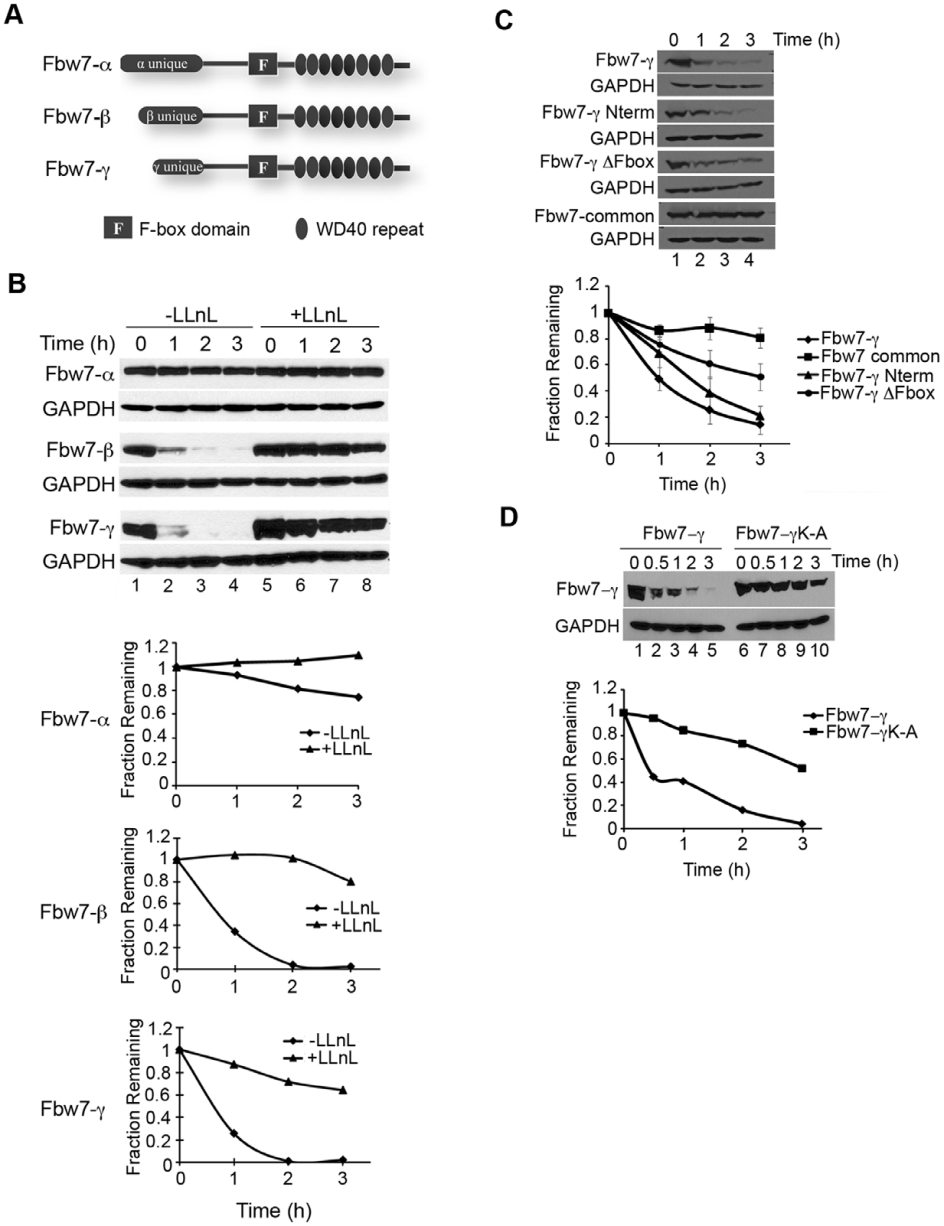
Fbw7-γ is an unstable protein and its proteolysis is dependent on the proteasome. A) Diagram of Fbw7 isoforms. The F-box motif and WD40 repeats are marked as shown. B) Fbw7-β and γ are unstable proteins, and their degradation is proteasome-dependent. Flag-tagged Fbw7 isoforms were expressed in HEK293T cells and protein stability assays were performed as described in Materials and Methods. Quantitation of a representative experiment is shown in the graph. C) The N-terminal unique region is critical for Fbw7-γ degradation. Protein stability assays were performed as described in (B) with cells expressing the indicated Flag-tagged proteins. Quantitation of three independent experiments is shown on the graph. Error bars indicate standard deviations. D) The lysine residues within the unique region of Fbw7-γ are critical for degradation. Protein stability assays were performed as described in (B) with cells expressing the indicated Flag-tagged proteins. Quantitation of a representative experiment is shown in the graph.

The Fbw7 isoforms (Figure 1A) exhibit differences in protein stability. Previous work indicated that the β and γ isoforms were unstable proteins, whereas the α isoform is stable [21]. We observe similar results in a protein stability assay using epitope-tagged Fbw7 constructs expressed in human HEK293T cells (Figure 1B, lanes 1–4). Briefly, cells expressing the indicated Fbw7 isoform were treated with the protein synthesis inhibitor cycloheximide and their turnover was monitored over time by immunoblotting. The turnover of the β and γ isoforms is inhibited when cells are also treated with a proteasome inhibitor, LLnL (Figure 1B, lanes 5–8), suggesting that they are targeted for proteasome-mediated degradation.

The Fbw7 isoforms arise from the use of a unique first exon, but are otherwise identical [22]. We chose to focus our studies on the degradation of Fbw7-γ, as this protein has proposed roles in targeting cyclin E and c-Myc for degradation in cancer cells [12], [24]. We reasoned that the N-terminal domain of Fbw7-γ might contribute to its protein stability characteristics. Thus, we examined the proteolysis of this domain compared to the full-length protein as well as mutants that lack the unique domain or the F-box region, respectively (Figure 1C). Both the unique N-terminal fragment of Fbw7-γ and the ΔF-box mutant exhibited partial stabilization of the protein compared to full-length Fbw7-γ, although the stabilization of the unique domain was relatively modest. By contrast, the Fbw7 common region, which contains the F-box motif but no unique sequence, was surprisingly stable. These results suggest that the N-terminal domain of Fbw7-γ plays an important role in the turnover of Fbw7-γ and that Fbw7-γ proteolysis is not fully controlled by an autoubiquitination mechanism that has been shown for other F-box proteins [28]. It also appears to be distinct from the recently described Plk2-mediated degradation of Fbw7, which requires phosphorylation of a residue in the common region to trigger degradation [29]. In addition, when the lysines in the γ isoform N-terminal fragment were mutated, the protein was significantly stabilized (Figure 1D), consistent with a model in which Fbw7-γ can be targeted for degradation by the ubiquitin proteasome pathway via the N-terminal domain.

### Identification of SLP-1 as an Fbw7-γ Specific Interaction Partner that Inhibits Fbw7-γ Turnover

**Figure 2:**
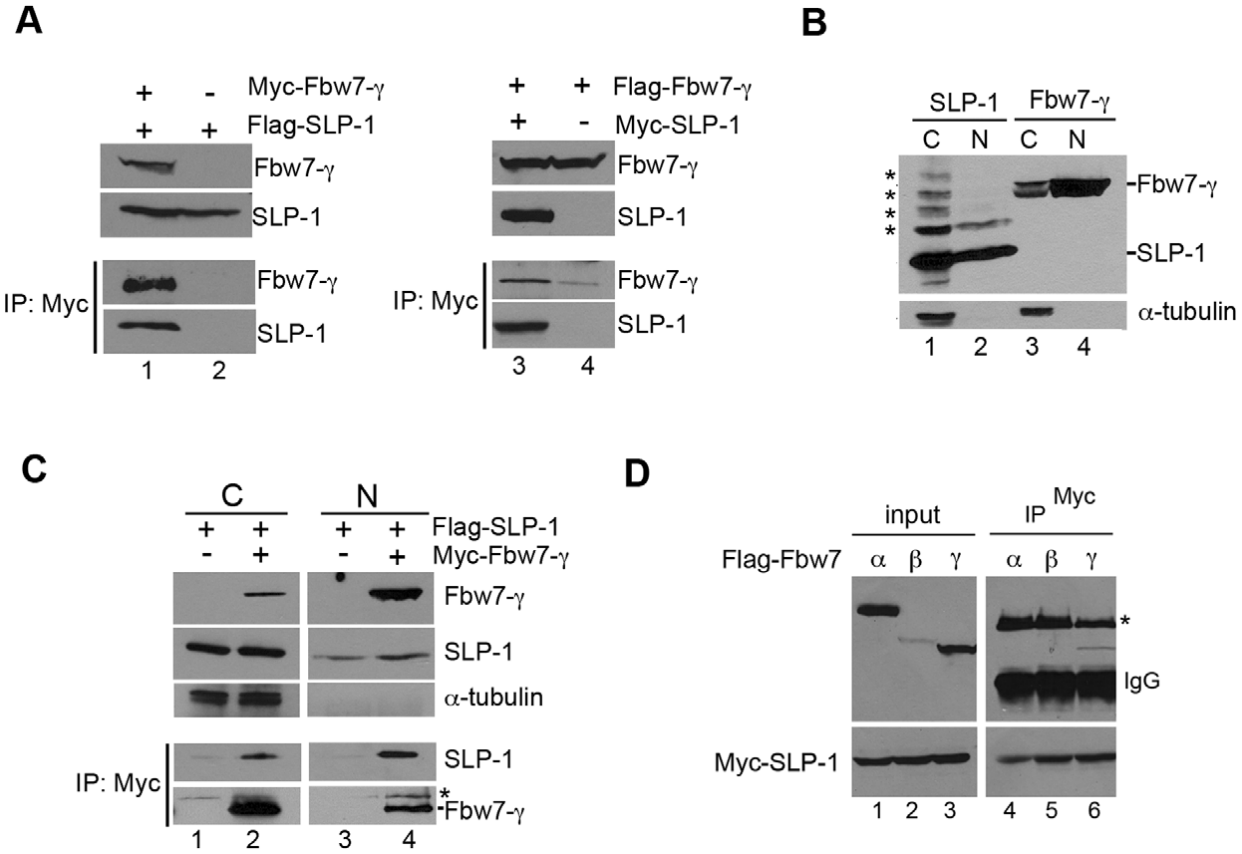
SLP1 is an Fbw7-γ-specific interacting protein. A) Fbw7-γ co-immunoprecipitates (co-IP) with SLP-1. Flag tagged SLP-1 was co-expressed with Myc-tagged Fbw7-γ (lane1) or vector (lane2) in HEK293T cells. Immunoprecipitation was performed as described in Materials and Methods. Reciprocal co-IP is shown in the right panel (lanes 3, 4). B) SLP-1 and Fbw7-γ co-fractionate. Standard fractionation assays from cells co-expressing Flag-tagged SLP-1 and Flag-tagged Fbw7-γ were performed as described in Materials and Methods. α-tubulin, a cytosolic protein, was used as a control. Asterisks indicate modified forms of SLP-1, C = cytosolic, N = nuclear. C) SLP-1 and Fbw7-γ co-immunoprecipitate in both nuclear and cytosolic fractions. Co-IPs were performed as in (A), except that cellular fractions from (B) were used. Asterisks indicate non-specific cross-reacting band. D) The Fbw7-γ and SLP-1 interaction is specific. Flag-tagged Fbw7 isoforms were co-expressed with myc-tagged SLP1. Immunoprecipitations were performed as in (A). IgG refers to heavy chain of the anti-Myc antibody, * indicates a non-specific band.

We hypothesized that regulation of Fbw7-γ protein stability would be controlled via binding partners that were able to recognize its N-terminus. To find interaction partners for Fbw7-γ, we performed a two-hybrid screen using the Fbw7-γ N-terminal domain as bait. We identified a novel interactor, SLP-1 (stomatin-like protein 1) using a HeLa cDNA library (data not shown). SLP-1 is a stomatin-like protein and is not well characterized [30]. Stomatins and stomatin-like proteins have been proposed to function in neuronal signaling in other systems [31], [32], but a function for SLP-1 has not been identified. The interaction between Fbw7-γ and SLP-1 was examined by reciprocal co-immunoprecipitation experiments (Figure 2A). In these experiments, Fbw7-γ and SLP-1 co-precipitated with each other. We examined the expression of each protein by indirect immunofluorescence microscopy to determine whether the proteins might indirectly interact by forming aggregates (Figure S1). Under these conditions, we did not observe the formation of aggregates, suggesting soluble Fbw7-γ and SLP-1 may interact with each other.

We next considered whether Fbw7-γ and SLP-1 might be co-localized. Our previous immunofluorescence experiment suggested that SLP-1 and Fbw7-γ might localize to both the nucleus and cytoplasm, at least when overexpressed. However, the intensity of the signal in either the nucleus or cytoplasm varied a bit depending on the construct used. We suspect this difference was related to expression levels or antibody efficiency as we consistently observed stronger signals with the Flag-tagged expression vectors. The localization of Fbw7-γ has been reported as either nuclear or nucleolar, depending on cell type [12], [21], whereas stomatin-like proteins are predicted to be cytoplasmic [33]. The Flag-tagged form of Fbw7-γ exhibited the strongest signal in the nucleus whereas Flag-tagged SLP-1 exhibited the strongest signal in the cytoplasm when localized by immunofluorescence, thus we reasoned that these proteins would provide a more stringent test for co-fractionation than the myc-tagged proteins. To examine this further, cells expressing Flag-tagged Fbw7-γ and Flag-tagged SLP-1 were fractionated into cytoplasmic and nuclear extracts and then probed for the presence of Fbw7-γ or SLP-1 (Figure 2B). We observed that Flag-tagged Fbw7-γ was found predominantly in the nuclear fraction but that there was a substantial cytoplasmic population as well (Figure 2B, lanes 3 and 4). Likewise, Flag-tagged SLP-1 was enriched in the cytoplasmic fraction, but retained a sizable population in the nucleus (Figure 2B, lanes 1 and 2). Strikingly, SLP-1 exhibited a ladder of higher molecular weight forms, which were most obvious in the cytoplasmic fraction. The nature of these modified forms remains to be determined. A cytoplasmic protein, alpha-tubulin, was used as a control for the quality of the fractionation. To determine whether Fbw7-γ and SLP-1 interact in either the nucleus or cytoplasm, we performed co-immunoprecipitations using nuclear and cytoplasmic extracts. As shown in Figure 2C, Flag-tagged SLP-1 and Myc-tagged Fbw7-γ co-precipitated in both nuclear and cytoplasmic fractions. Together, these results suggest that at least a fraction of the Fbw7-γ and SLP-1 populations are likely to co-localize. However, because these proteins are overexpressed, we cannot determine whether they are more likely to interact in the nucleus or cytoplasm at physiological levels from these results.

To examine whether the interaction between SLP-1 and Fbw7-γ was specific, we performed co-immunoprecipitation experiments with Flag-tagged Fbw7-α and Fbw7-β isoforms co-expressed with myc-tagged SLP-1, using Fbw7-γ as a control (Figure 2D). In this experiment, we observed Fbw7-γ co-precipitation with SLP-1 but neither Fbw7-α nor Fbw7-β co-precipitated with SLP-1. However, the expression of Fbw7-β was very weak relative to Fbw7-α and Fbw7-γ, so we cannot rule out the possibility that Fbw7-β might be able to interact with SLP-1, but that we were unable to detect it. Nevertheless, the absence of an interaction with Fbw7-α suggests that SLP-1 is likely to show some specificity in interacting with Fbw7 isoforms.

**Figure 3:**
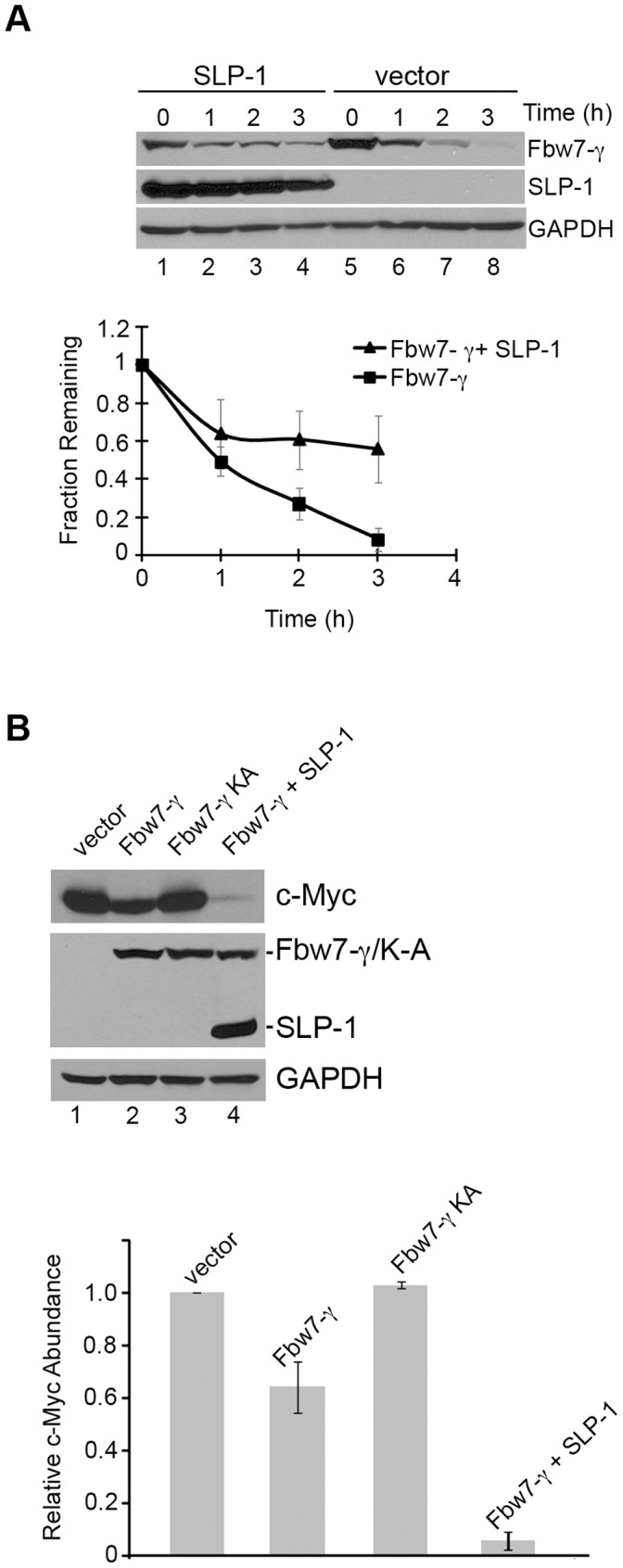
A) SLP-1 overexpression inhibits Fbw7-γ turnover. Flag-tagged Fbw7-γ was co-transfected with Flag-tagged SLP-1 (lane 1–4) or empty vector (lane 5–8) into HEK293T cells and protein stability assays were performed as described in Materials and Methods. Quantitation of three independent experiments is shown on the graph. Error bars indicate standard deviations. B) Abundance of co-expressed c-Myc is decreased in cells expressing Flag-tagged SLP-1 and Flag-tagged Fbw7-γ. HEK293T cells were transfected with the indicated expression constructs and cell extracts probed with anti-Flag, anti-Myc (9E10) or anti-GAPDH antibodies. Quantitation of three independent experiments is shown on the graph. Error bars indicate standard deviations.

To determine whether the binding of SLP-1 to Fbw7-γ might have an effect on Fbw7-γ protein turnover, we performed protein stability assays in HEK293T cells overexpressing these proteins. When SLP-1 and Fbw7-γ are co-overexpressed, Fbw7-γ turnover was inhibited in a cycloheximide-based stability assay, increasing the Fbw7-γ 60-minute half-life at least three-fold (Figure 3A). This observation suggests that it is possible for the binding of SLP-1 to Fbw7-γ to affect the regulation of the Fbw7-γ protein in transformed cells.

To examine whether the delay in Fbw7-γ turnover caused by overexpression of SLP-1 might affect Fbw7-γ SCF E3 ligase function, we examined the abundance of c-Myc, a protein thought to be an ubiquitination target of Fbw7-γ [12], [34]. In cells expressing Fbw7-γ and c-Myc, the abundance of c-Myc was decreased compared to cells expressing a control vector (Figure 3B), as expected based on previous observations [12]. When SLP-1 was co-expressed with Fbw7-γ, we observed an even greater decrease in the abundance of c-Myc, suggesting that SLP-1 may protect Fbw7-γ from degradation and that stabilized Fbw7-γ can still assemble a functional SCF complex. However, expression of the Fbw7^K-A^ mutant had little effect on c-Myc abundance and the explanation for such a result is not clear. It is possible that this mutant may be compromised in SCF function as well as protein turnover.

### Cdk2 Interacts with Both Fbw7-γ and SLP1

**Figure 4:**
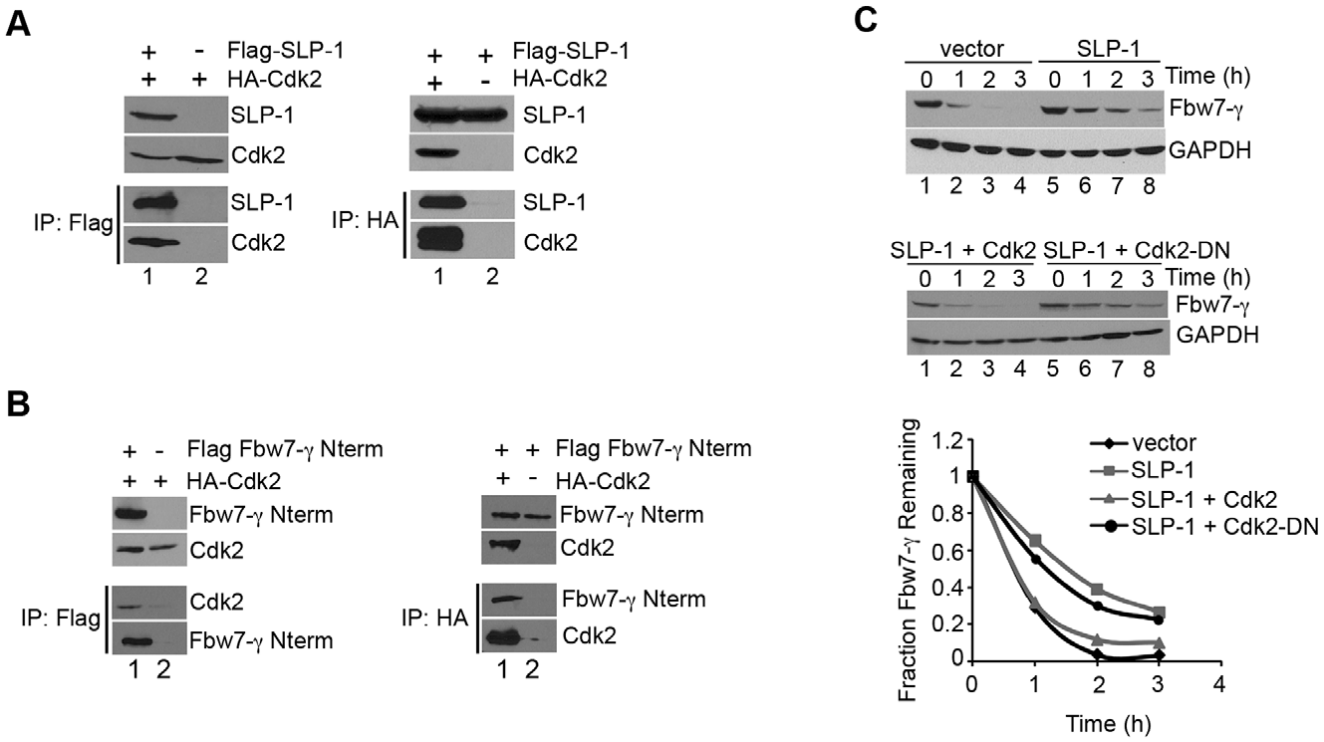
SLP-1 and Fbw7-γ interact with Cdk2. A) SLP-1 interacts with Cdk2. HA-tagged Cdk2 was transfected with Flag-tagged SLP-1 or empty vector into HEK293T cells. Co-immunoprecipitations were performed as described in Materials and Methods. B) Cdk2 interacts with Fbw7-γ. Co-IPs were performed as in (A) in cells expressing the indicated proteins. C) Co-expression of Cdk2 and SLP-1 promotes turnover of Flag-tagged Fbw7-γ. Protein stability assays were performed as described in Materials and Methods in cells expressing the indicated proteins. Quantitation of a representative experiment is shown in the graph.

We considered whether other proteins that interact with Fbw7-γ might also interact with SLP-1. One candidate we tested was Cdk2, which partners with cyclin E, a substrate of the SCF^Fbw7^ complex. Previous work suggested that Cdk2 activity can inhibit other E3 ubiquitin ligases by targeting them for degradation [35]. To test whether SLP-1 interacted with Cdk2, we performed immunoprecipitations using protein extracts from HEK293T cells expressing epitope-tagged versions of Cdk2, Fbw7-γ and SLP-1. As shown in Figure 4A, Cdk2 and SLP-1 co-immunoprecipitated and we find that the interaction was observed in reciprocal co-immunoprecipitations. We also determined that overexpression of HA-tagged Cdk2 did not lead to the formation of aggregates using immunofluorescence microscopy (Figure S1). Since Cdk2 likely interacts with Fbw7-γ as part of a complex with cyclin E as cyclin E is being targeted for ubiquitination, we performed co-immunoprecipitations from cells expressing only the unique N-terminus of Fbw7-γ, as Fbw7 substrates bind the WD40 domain found in the C-terminal portion of the protein, a region common to all Fbw7 isoforms [5], [6]. Under these conditions, we observed co-immunoprecipitation of Cdk2 and the Fbw7-γ N-terminal domain (Figure 4B). Together, our results indicate that Cdk2 can interact with both SLP-1 and Fbw7-γ, although we cannot determine whether all three proteins are in a complex simultaneously from these data.

As Cdk2 was able to interact with Fbw7-γ and SLP-1, we sought to determine whether Cdk2 overexpression might alter the inhibitory effect of SLP-1 overexpression on Fbw7-γ degradation. When both Cdk2 and SLP-1 were overexpressed, Fbw7-γ was turned over as efficiently as when Fbw7-γ was expressed with vector alone (Figure 4C, top panel and bottom graph). The effect of Cdk2 was dependent on its kinase activity as Fbw7-γ turnover when SLP-1 and the Cdk2 kinase-dead mutant are co-overexpressed mimicked the degradation rate observed when just SLP-1 was overexpressed (Figure 4C, middle panel and bottom graph). These results suggest that SLP-1 and Cdk2 may have opposing functions in regulating Fbw7-γ degradation.

## Discussion

We have identified two interaction partners for Fbw7-γ that can affect Fbw7-γ degradation when overexpressed in transformed cells. The identification of SLP-1 as an interaction partner for Fbw7-γ is novel, as there was previously no evidence of stomatin family members interacting with SCF ubiquitin ligases in human cells or other systems. SLP-1 belongs to the SPFH (stomatins/prohibitins/flotillins/Hf/K/C) superfamily, which is highly conserved but little functional data exists for many family members [36]. SLP-1 is conserved from invertebrates to humans [30], [36] and the *C. elegans* homolog of SLP-1, *unc-24*, is proposed to have a role in neural function [31], [32]. Interestingly, human SLP-1 mRNA expression is highest in neuronal tissue as is Fbw7-γ mRNA expression [22], [30], indicating that the proteins are likely expressed in the same type of cells and that SLP-1 might have a role in protecting Fbw7-γ from degradation in neuronal cells. Future studies will be necessary to determine whether Fbw7-γ and SLP-1 interact in non-transformed cells and whether the interaction is important at the organismal level.

Our results indicate that Fbw7-γ is an unstable protein, targeted for destruction by the proteasome. It is not known which E2/E3 complex controls Fbw7-γ ubiquitination. Our experiments suggest that the ubiquitin-mediated degradation of Fbw7-γ is not fully controlled by an autocatalytic mechanism, as has been observed with some F-box proteins [28], because the unique N-terminal domain is also important for turnover. In addition, deletion of the F-box domain from Fbw7-γ does not fully stabilize the protein, as would be expected for an autocatalytic means of destruction. We look forward to future studies that might identify the pathway responsible for Fbw7-γ turnover.

Our studies suggest that the binding of SLP-1 to the N-terminus of Fbw7-γ does not interfere with the assembly of a functional SCF^Fbw7-γ^ complex in transformed cells, as c-Myc appears to still be targeted for degradation when both SLP-1 and Fbw7-γ are expressed. Further, SLP-1-dependent stabilization of Fbw7-γ leads to an even greater reduction in c-Myc abundance than when Fbw7-γ is expressed alone. One explanation for our results is that since Fbw7-γ is stabilized, there are more functional SCF^Fbw7-γ^ complexes available to target c-Myc for ubiquitination. Alternatively, it may be that SLP-1 inhibits Fbw7-γ turnover because it is a co-factor for the SCF ubiquitin ligase complex with a particular substrate protein. Such co-factors have been identified with other SCF-type complexes, such as Cks1, which is required for the SCF^Skp2^- mediated ubiquitination of p27 [37], [38]. How SLP-1 inhibits Fbw7-γ turnover is an open question, but it seems likely that it could be via physically blocking access to the N-terminal domain of Fbw7-γ, which we show to be required for turnover. Regardless of the mechanism involved in inhibiting Fbw7-γ turnover, as c-Myc is a proto-oncogene and is often overexpressed or amplified in tumor cells [39], an intriguing possibility to control c-Myc protein levels might involve regulation of the abundance of Fbw7-γ and SLP-1.

Fbw7-γ and SLP-1 co-precipitate with Cdk2 in transformed cells, but is not clear whether Cdk2 phosphorylates either of these proteins. SLP-1 contains two consensus CDK sites but Fbw7-γ does not contain any CDK consensus sites in the unique N-terminal domain (W. Zhang and D. M. Koepp, unpublished observations). The mechanism by which co-expression of Cdk2 might inhibit the effect of SLP-1 expression on Fbw7-γ turnover is not known. One possibility is that Cdk2 outcompetes SLP-1 for binding the N-terminus of Fbw7-γ. In this scenario, Cdk2 binding to the N-terminus of Fbw7-γ would not interfere with Fbw7-γ protein turnover. Alternatively, Cdk2 may affect SLP-1 directly to prevent it from inhibiting Fbw7-γ degradation. Future studies will be required to distinguish between these possibilities.

Overall, these studies identify new protein partners of Fbw7-γ and suggest a regulatory pathway exists for degradation of the Fbw7-γ protein.

## Supporting Information

Figure S1 Overexpression of epitope-tagged SLP-1, Fbw7-γ and Cdk2 does not result in aggregate formation. Cells expressing the indicated tagged proteins were prepared for indirect immunofluorescence microscopy as described in Materials and Methods. The indicated proteins were detected using anti-Flag, anti-Myc or anti-HA antibodies followed by FITC-conjugated secondary antibodies. DAPI was used to localize DNA.
(DOC)

## References

[1] Welcker M, Clurman BE (2008) FBW7 ubiquitin ligase: a tumour suppressor at the crossroads of cell division, growth and differentiation. Nature Reviews Cancer 8: 83–93. 18094723 10.1038/nrc2290

[2] Tetzlaff MT, Yu W, Li M, Zhang P, Finegold M, et al (2004) Defective cardiovascular development and elevated cyclin E and Notch proteins in mice lacking the Fbw7 F-box protein. Proc Natl Acad Sci U S A 101: 3338–3345. 14766969 10.1073/pnas.0307875101 PMC373463

[3] Tsunematsu R, Nakayama K, Oike Y, Nishiyama M, Ishida N, et al (2004) Mouse Fbw7/Sel-10/Cdc4 is required for notch degradation during vascular development. J Biol Chem 279: 9417–9423. 14672936 10.1074/jbc.M312337200

[4] Mao JH, Perez-Losada J, Wu D, Delrosario R, Tsunematsu R, et al (2004) Fbxw7/Cdc4 is a p53-dependent, haploinsufficient tumour suppressor gene. Nature 432: 775–779. 15592418 10.1038/nature03155

[5] Koepp DM, Schaefer LK, Ye X, Keyomarsi K, Chu C, et al (2001) Phosphorylation-dependent ubiquitination of cyclin E by the SCFFbw7 ubiquitin ligase. Science 294: 173–177. 11533444 10.1126/science.1065203

[6] Strohmaier H, Spruck CH, Kaiser P, Won KA, Sangfelt O, et al (2001) Human F-box protein hCdc4 targets cyclin E for proteolysis and is mutated in a breast cancer cell line. Nature 413: 316–322. 11565034 10.1038/35095076

[7] Moberg KH, Bell DW, Wahrer DC, Haber DA, Hariharan IK (2001) Archipelago regulates Cyclin E levels in Drosophila and is mutated in human cancer cell lines. Nature 413: 311–316. 11565033 10.1038/35095068

[8] Wu G, Lyapina S, Das I, Li J, Gurney M, et al (2001) SEL-10 is an inhibitor of notch signaling that targets notch for ubiquitin-mediated protein degradation. Mol Cell Biol 21: 7403–7415. 11585921 10.1128/MCB.21.21.7403-7415.2001 PMC99913

[9] Oberg C, Li J, Pauley A, Wolf E, Gurney M, et al (2001) The Notch intracellular domain is ubiquitinated and negatively regulated by the mammalian Sel-10 homolog. J Biol Chem 276: 35847–35853. 11461910 10.1074/jbc.M103992200

[10] Gupta-Rossi N, Le Bail O, Gonen H, Brou C, Logeat F, et al (2001) Functional interaction between SEL-10, an F-box protein, and the nuclear form of activated Notch1 receptor. J Biol Chem 276: 34371–34378. 11425854 10.1074/jbc.M101343200

[11] Nateri AS, Riera-Sans L, Da Costa C, Behrens A (2004) The ubiquitin ligase SCFFbw7 antagonizes apoptotic JNK signaling. Science 303: 1374–1378. 14739463 10.1126/science.1092880

[12] Welcker M, Orian A, Grim JE, Eisenman RN, Clurman BE (2004) A nucleolar isoform of the Fbw7 ubiquitin ligase regulates c-Myc and cell size. Curr Biol 14: 1852–1857. 15498494 10.1016/j.cub.2004.09.083

[13] Yada M, Hatakeyama S, Kamura T, Nishiyama M, Tsunematsu R, et al (2004) Phosphorylation-dependent degradation of c-Myc is mediated by the F-box protein Fbw7. EMBO J 23: 2116–2125. 15103331 10.1038/sj.emboj.7600217 PMC424394

[14] Mao JH, Kim IJ, Wu D, Climent J, Kang HC, et al (2008) FBXW7 targets mTOR for degradation and cooperates with PTEN in tumor suppression. Science 321: 1499–1502. 18787170 10.1126/science.1162981 PMC2849753

[15] Sundqvist A, Bengoechea-Alonso MT, Ye X, Lukiyanchuk V, Jin JP, et al (2005) Control of lipid metabolism by phosphorylation-dependent degradation of the SREBP family of transcription factors by SCFFbw7. Cell Metabolism 1: 379–391. 16054087 10.1016/j.cmet.2005.04.010

[16] Punga T, Bengoechea-Alonso MT, Ericsson J (2006) Phosphorylation and ubiquitination of the transcription factor sterol regulatory element-binding protein-1 in response to DNA binding. Journal of Biological Chemistry 281: 25278–25286. 16825193 10.1074/jbc.M604983200

[17] Olson BL, Hock MB, Ekholm-Reed S, Wohlschlegel JA, Dev KK, et al (2008) SCFCdc4 acts antagonistically to the PGC-1 alpha transcriptional coactivator by targeting it for ubiquitin-mediated proteolysis. Genes & Development 22: 252–264. 18198341 10.1101/gad.1624208 PMC2192758

[18] Won KA, Reed SI (1996) Activation of cyclin E/CDK2 is coupled to site-specific autophosphorylation and ubiquitin-dependent degradation of cyclin E. EMBO J. 15: 4182–4193. PMC452142 8861947

[19] Orlicky S, Tang X, Willems A, Tyers M, Sicheri F (2003) Structural basis for phosphodependent substrate selection and orientation by the SCFCdc4 ubiquitin ligase. Cell 112: 243–256. 12553912 10.1016/s0092-8674(03)00034-5

[20] Hao B, Oehlmann S, Sowa ME, Harper JW, Pavletich NP (2007) Structure of a Fbw7-Skp1-cyclin E complex: Multisite-phosphorylated substrate recognition by SCF ubiquitin ligases. Molecular Cell 26: 131–143. 17434132 10.1016/j.molcel.2007.02.022

[21] Grim JE, Gustafson MP, Hirata RK, Hagar AC, Swanger J, et al (2008) Isoform- and cell cycle-dependent substrate degradation by the Fbw7 ubiquitin ligase. Journal of Cell Biology 181: 913–920. 18559665 10.1083/jcb.200802076 PMC2426948

[22] Spruck CH, Strohmaier H, Sangfelt O, Muller HM, Hubalek M, et al (2002) hCDC4 gene mutations in endometrial cancer. Cancer Res 62: 4535–4539. 12183400

[23] Popov N, Wanzel M, Madiredjo M, Zhang D, Beijersbergen R, et al (2007) The ubiquitin-specific protease USP28 is required for MYC stability. Nature Cell Biology 9: 765–U771. 17558397 10.1038/ncb1601

[24] van Drogen F, Sangfelt O, Malyukova A, Matskova L, Yeh E, et al (2006) Ubiquitylation of cyclin E requires the sequential function of SCF complexes containing distinct hCdc4 isoforms. Mol Cell 23: 37–48. 16818231 10.1016/j.molcel.2006.05.020

[25] Sangfelt O, Cepeda D, Malyukova A, van Drogen F, Reed SI (2008) Both SCFCdc4 alpha and SCFCdc4 gamma are required for cyclin E turnover in cell lines that do not overexpress cyclin E. Cell Cycle. 7: 1075–1082. 10.4161/cc.7.8.5648 18414042

[26] Zhang W, Koepp DM (2006) Fbw7 isoform interaction contributes to cyclin E proteolysis. Mol Cancer Res 4: 935–943. 17189384 10.1158/1541-7786.MCR-06-0253

[27] Bai C, Elledge SJ (1996) Gene identification using the yeast two-hybrid system. Methods Enzymol 273: 331–347. 8791622 10.1016/s0076-6879(96)73029-x

[28] Zhou P, Howley PM (1998) Ubiquitination and degradation of the substrate recognition subunits of SCF ubiquitin-protein ligases. Mol Cell 2: 571–580. 9844630 10.1016/s1097-2765(00)80156-2

[29] Cizmecioglu O, Krause A, Bahtz R, Ehret L, Malek N, et al (2012) Plk2 regulates centriole duplication through phosphorylation-mediated degradation of Fbxw7 (human Cdc4). J Cell Sci 125: 981–992. 22399798 10.1242/jcs.095075

[30] Seidel G, Prohaska R (1998) Molecular cloning of hSLP-1, a novel human brain-specific member of the band 7 MEC-2 family similar to Caenorhabditis elegans UNC-24. Gene 225: 23–29. 9931417 10.1016/s0378-1119(98)00532-0

[31] Sedensky MM, Siefker JM, Koh JY, Miller DM, Morgan PG (2004) A stomatin and a degenerin interact in lipid rafts of the nervous system of Caenorhabditis elegans. American Journal of Physiology-Cell Physiology 287: C468–C474. 15102610 10.1152/ajpcell.00182.2003

[32] Zhang SF, Arnadottir J, Keller C, Caldwell GA, Yao CA, et al (2004) MEC-2 is recruited to the putative mechanosensory complex in C-elegans touch receptor neurons through its stomatin-like domain. Current Biology 14: 1888–1896. 15530389 10.1016/j.cub.2004.10.030

[33] Mairhofer M, Steiner M, Salzer U, Prohaska R (2009) Stomatin-like Protein-1 Interacts with Stomatin and Is Targeted to Late Endosomes. Journal of Biological Chemistry 284: 29218–29229. 19696025 10.1074/jbc.M109.014993 PMC2781465

[34] Bonetti P, Davoli T, Sironi C, Amati B, Pelicci PG, et al (2008) Nucleophosmin and its AML-associated mutant regulate c-Myc turnover through Fbw7 gamma. J Cell Biol 182: 19–26. 18625840 10.1083/jcb.200711040 PMC2447890

[35] Hayami R, Sato K, Wu W, Nishikawa T, Hiroi J, et al (2005) Down-regulation of BRCA1-BARD1 ubiquitin ligase by CDK2. Cancer Res 65: 6–10. 15665273

[36] Green JB, Young JPW (2008) Slipins: ancient origin, duplication and diversification of the stomatin protein family. BMC Evolutionary Biology 8: -. 10.1186/1471-2148-8-44 PMC2258279 18267007

[37] Ganoth D, Bornstein G, Ko TK, Larsen B, Tyers M, et al (2001) The cell-cycle regulatory protein Cks1 is required for SCF(Skp2)-mediated ubiquitinylation of p27. Nat Cell Biol 3: 321–324. 11231585 10.1038/35060126

[38] Spruck C, Strohmaier H, Watson M, Smith AP, Ryan A, et al (2001) A CDK-independent function of mammalian Cks1: targeting of SCF(Skp2) to the CDK inhibitor p27Kip1. Mol Cell 7: 639–650. 11463388 10.1016/s1097-2765(01)00210-6

[39] Brooks TA, Hurley LH (2009) The role of supercoiling in transcriptional control of MYC and its importance in molecular therapeutics. Nat Rev Cancer 9: 849–861. 19907434 10.1038/nrc2733

[40] Ye X, Nalepa G, Welcker M, Kessler BM, Spooner E, et al (2004) Recognition of phosphodegron motifs in human cyclin E by the SCF(Fbw7) ubiquitin ligase. J Biol Chem 279: 50110–50119. 15364936 10.1074/jbc.M409226200

[41] van den Heuvel S, Harlow E (1993) Distinct roles for cyclin-dependent kinases in cell cycle control. Science 262: 2050–2054. 8266103 10.1126/science.8266103

