# Supplementary material for: The Stomatin-Like Protein SLP-1 and Cdk2 Interact with the F-Box Protein Fbw7-γ
Source: PLoS One. 2012 Oct 17;7(10):e47736. doi: 10.1371/journal.pone.0047736 (PMC3474722; doi:10.1371/journal.pone.0047736)
Supplement: Figure S1 — Overexpression of epitope-tagged SLP-1, Fbw7-γ and Cdk2 does not result in aggregate formation. Cells expressing the indicated tagged proteins were prepared for indirect immunofluorescence microscopy as described in Materials and Methods. The indicated proteins were detected using anti-Flag, anti-Myc or anti-HA antibodies followed by FITC-conjugated secondary antibodies. DAPI was used to localize DNA. (DOC) [file pone.0047736.s001.doc]

**Supporting Information – Figure S1 and Legend**

**
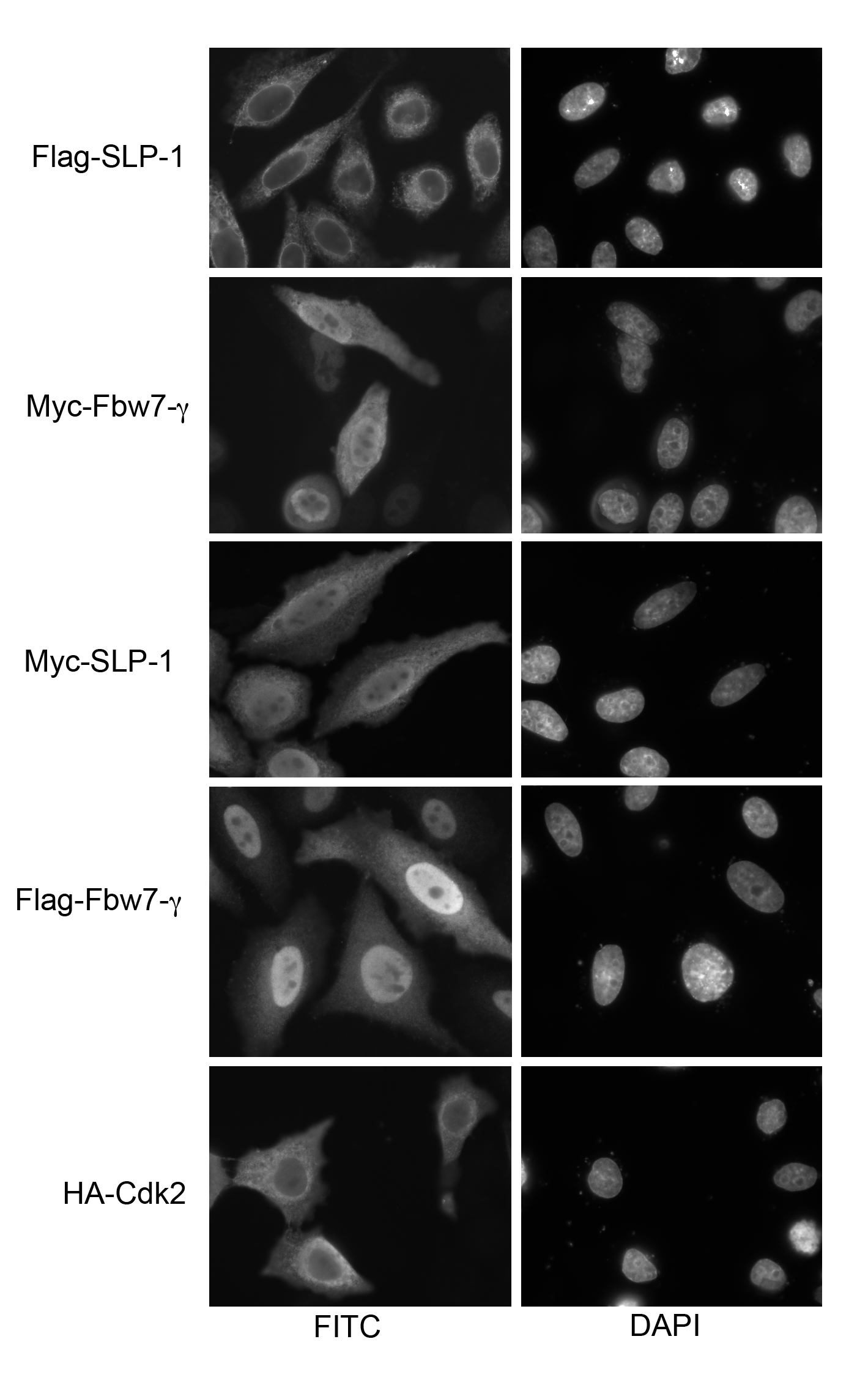
**

**Supplemental Figure S1**. Overexpression of epitope-tagged SLP-1, Fbw7-γ and Cdk2 does not result in aggregate formation. Cells expressing the indicated tagged proteins were prepared for indirect immunofluorescence microscopy as described in Materials and Methods. The indicated proteins were detected using anti-Flag, anti-Myc or anti-HA antibodies followed by FITC-conjugated secondary antibodies. DAPI was used to localize DNA.
